# Toll like-receptor agonist Pam_3_Cys modulates the immunogenicity of liposomes containing the tuberculosis vaccine candidate H56

**DOI:** 10.1007/s00430-020-00657-3

**Published:** 2020-02-04

**Authors:** Kathrin Kennerknecht, Reiner Noschka, Florian Löffler, Stephanie Wehrstedt, Gabriel Kristian Pedersen, Daniel Mayer, Mark Grieshober, Dennis Christensen, Steffen Stenger

**Affiliations:** 1 Institut für Medizinische Mikrobiologie und Hygiene, Universitätsklinikum Ulm, Albert-Einstein-Allee 23, 89081 Ulm, Germany; 2 Department of Infectious Disease Immunology, Statens Serum Institut, Artillerivej 5, 2300 Copenhagen S, Denmark

**Keywords:** Tuberculosis, Vaccination, Liposomes, Toll like-receptor agonist

## Abstract

A major roadblock in the development of novel vaccines is the formulation and delivery of the antigen. Liposomes composed of a dimethyldioctadecylammonium (DDA) backbone and the adjuvant trehalose-6-6-dibehenate (TDB, termed “cationic adjuvant formulation (CAF01)”, promote immunogenicity and protective efficacy of vaccines, most notably against infection with *Mycobacterium tuberculosis*. Specifically, the multicomponent antigen H56 delivered by CAF01 protects against tuberculosis in mice. Here we investigated whether the inclusion of immune-modulatory adjuvants into CAF01 modulates the immunogenicity of H56/CAF01 in vitro and in vivo. Based on our recent findings we selected the active sequence of the mycobacterial 19 kDa lipoprotein, Pam_3_Cys, which interacts with Toll like receptor 2 to induce an antimicrobial pathway. H56/CAF01-Pam_3_Cys liposomes were characterized for Pam_3_Cys incorporation, size, toxicity and activation of primary human macrophages. Macrophages efficiently take up H56/CAF01-Pam_3_Cys and trigger the release of significantly higher levels of TNF, IL-12 and IL-10 than H56/CAF01 alone. To evaluate the immunogenicity in vivo, we immunized mice with H56/CAF01-Pam_3_Cys and measured the release of IFN-γ and IL-17A by lymph node cells and spleen cells. While the antigen-specific production of IFN-γ was reduced by inclusion of Pam_3_Cys into H56/CAF01, the levels of IL-17A remained unchanged. In agreement with this finding, the concentration of the IFN-γ-associated IgG2a antibodies in the serum was lower than in H56/CAF01 immunized animals. These results provide proof of concept that Toll like-receptor agonist can be included into liposomes to modulate immune responses. The discordant results between the in vitro studies with human macrophages and in vivo studies in mice highlight the relevance and complexity of comparing immune responses in different species

## Introduction

Vaccination is the most efficient strategy to prevent infectious diseases. Vaccination against several viral infections and toxin-mediated bacterial diseases revolutionized the prevention of severe infections. In contrast, vaccination against infections, which require the induction of T-cell-mediated immunity has proven difficult. The obstacles in designing appropriate vaccines include the delivery of the vaccine antigen into the antigen-presenting cell, the loading on MHC and CD1 molecules, the recognition by naïve T cells and the induction of long-lasting memory cells, which elicit a protective immune response upon encounter with the pathogen. One approach to bias the immune response towards protective cell-mediated immunity is to formulate the vaccine antigen into adjuvants. The cationic adjuvant formulation (CAF) platform promotes Th1-polarization and is applied for more than a decade to formulate vaccine antigens against infections, in which Th1-mediated effector mechanisms are critical for protection [1]. CAF is composed of the quaternary ammoniumsurfactant *N*,*N*-dimethyl-*N*,*N*-dioctadecylammonium (DDA) formulated into liposomes. The combination of DDA with the synthetic cord factor analogue trehalose 6,6-dibehenate (TDB) promotes protective immune responses without enhancing toxicity [2]. This adjuvant−termed CAF01−is stable, polarizes the T-cell response towards Th1/Th17 and activates macrophages by binding to MINCLE and triggering SYK/Card9/Bcl10/Malt-1 signaling [3]. The potential of CAF01 as an adjuvant was demonstrated with the anti-tuberculosis subunit vaccine H56, which is a fusion protein of the mycobacterial antigens Ag85B, ESAT-6 and Rv2660c. H56:CAF01 elicits a Th1/Th17-CD4 T-cell response in the lung of *Mycobacterium tuberculosis* (*Mtb*) infected mice and provides prolonged control of infection in mice [4]. The CAF01 adjuvant system offers an ideal platform to include additional immune-stimulators to skew the immune response into the desired direction [2].

The mycobacterial 19 kDa lipoprotein is a cell wall associated lipoprotein expressed in mycobacteria including *Mtb* [5]. The 19 kDa lipoprotein interaction with toll like receptors 2/1 on human macrophages to induce pleiotropic biological effects, including the induction of apoptosis, the induction of IL-12 release to the inhibition of antigen presentation [6–9]. Pam_3_Cys, the active site of the 19 kDa lipoprotein activates an antimicrobial pathway in human macrophages that involves binding to TLR2/1, the upregulation of the vitamin D receptor and the induction of the antimicrobial peptide cathelicidin ultimately resulting in killing of intracellular *Mtb* [10]. Based on the capability of CAF01 to promote Th1/Th17 responses and the ability of Pam_3_Cys to induce an antimicrobial pathway we tested the hypothesis, that the inclusion of Pam_3_Cys into liposome-based CAF01 enhances immune responses in vitro using primary human macrophages and in vivo by immunizing mice.

## Materials and methods

### Generation of liposomes

CAF01 liposomes were generated via lipid film hydration as described previously [11]. Pam_3_Cys (EMC Microcollections, Tübingen, Germany) was dissolved together with the CAF01 components (5:1, w/w; Avanti Polar Lipids, Alabaster, AL) in chloroform (VWR, Radnor, PA): methanol (Sigma-Aldrich, St. Louis, MO) (9:1, v/v). The organic solvent was evaporated under nitrogen flow forming a lipid film at the bottom of a glass vial. Liposomal vesicles were formed by hydrating the lipid film in 10 mM Tris-buffer (pH 7.4; Sigma-Aldrich, St. Louis, MO) for 25 min at 10 °C above the main phase transition of DDA (*T*_m_ ≈ 47 °C) with in-between mixing. The final concentrations of DDA and TDB were 1.25 mg/ml and 0.25 mg/ml, respectively. The final concentrations of Pam_3_Cys were 0.1 mg/ml, 0.2 mg/ml or 0.4 mg/ml. The fusion protein H56 (Statens Serum Institute, Copenhagen, Denmark) [12] was diluted in Tris-buffer (10 mM, pH 7.4) and added in a 1:1 mixture to the hydrated liposomes at a final concentration of 0.1 mg/ml. Fluorescently-labeled liposomes were prepared by adding 0.2 mol% 1,1ʹ-dioctadecyl-3,3,3ʹ,3ʹ-tetramethylindo-carbocyanine perchlorate (DilC; Sigma-Aldrich, St. Louis, MO) to the lipids in organic solvent.

### Nanoparticle tracking analysis

The vesicle size of liposomes diluted 1:100 in Tris-buffer (10 mM, pH 7.4) was determined by Nanoparticle Tracking Analysis (NTA) using NanoSight LM10 (Malvern Panalytical, Malvern, UK). NanoSight NTA software 2.3 (Malvern Panalytical, Malvern, UK) was used for data acquisition and analysis.

### Differential scanning calorimetry analysis

The heat capacity of the liposomes was determined using a Micro DSCIII CSEvol (Setaram). 800 μl of undiluted sample was loaded in a standard Hilleroy metal cell. The control cell was filled with the same weight of 10 mM Tris buffer (pH 7.4). Samples were heated from 20 to 80 °C at a scanning rate of 0.5 °C/min and thermograms were obtained and analyzed using supplied software. The first of two scans of each sample was used for data analysis.

### Cell culture and generation of antigen-presenting cells

Buffy coats from healthy donors were provided by the German Red Cross located at the Institute of Transfusion Medicine (Ulm University, Ulm, Germany). Primary human cells were cultured in RPMI 1640 medium (Life Technologies, Carlsbad, CA) supplemented with glutamine (2 mM; Sigma‐Aldrich, St. Louis, MO), 10 mM HEPES, 100 µg/ml streptomycin, 60 μg/ml penicillin (all Biochrom, Berlin, Germany), and 5% heat‐inactivated human AB serum (Sigma‐Aldrich, St. Louis, MO). CD1^+^ antigen-presenting cells (APCs) were generated by incubating plastic adherent peripheral blood mononuclear cells (PBMCs) for four days with IL-4 (10 ng/ml; BioLegend, San Diego, CA) and granulocyte–macrophage colony-stimulating factor (GM-CSF) (10 ng/ml; Miltenyi Biotec, Bergisch Gladbach, Germany). CD1^+^ antigen presenting cells were used, because the primary objective of this work is the improvement of the presentation of mycobacterial lipid antigens, which are presented by group 1 CD1 molecules not expressed by conventional monocytes or macrophages.

### Annexin V-FITC staining

The percentage of viable cells was determined by Annexin V/propidium iodide (PI) staining using the “FITC Annexin V Apoptosis Detection Kit I” from BD Biosciences (Franklin Lakes, NJ). The manufacturer's protocol was followed, data was recorded using a FACSCalibur^™^ (BD Biosciences) and analyzed using FlowJo 10.4.1. software (Tree Star Inc).

### Confocal laser scanning microscopy

Intracellular localization of liposomes within CD1^+^ APCs was investigated by confocal microscopy. 0.1 × 10^6^ CD1^+^ APCs were seeded in 200 µl cell culture medium in an eight-chamber slide (Thermo Fisher Scientific, Waltham, MA) and incubated overnight with fluorescently-labeled liposomes. Cells were fixed with 4% paraformaldehyde (Sigma-Aldrich, St. Louis, MO), permeabilized for 10 min in 0.5% bovine serum albumin (BSA), 0.1% Triton X-100 and 0.05% Tween 20 (all Sigma-Aldrich, St. Louis, MO) diluted in phosphate buffered saline (PBS) (Life Technologies, Carlsbad, CA). Samples were blocked for one hour with blocking buffer (1% BSA, 0.1% Triton X-100 in PBS) and incubated at room temperature for one hour with primary antibodies directed against early endosomes (rabbit polyclonal early endosome antigen 1 (EEA1); 1:1000; Abcam, Cambridge, UK) or lysosomes (rabbit polyclonal lysosomal-associated membrane protein 1 (LAMP1); 1:200; Abcam, Cambridge, UK). After washing three times, cells were incubated with the secondary antibody Cy2-conjugated donkey anti-rabbit IgG (1:100; Dianova, Hamburg, Germany) for one hour at room temperature. Cell nuclei were stained with 1 µg/ml 4ʹ,6-diamidino-2-phenylindole (DAPI; Sigma-Aldrich, St. Louis, MO) for 10 min and slides were mounted with aquatex (Merck, Darmstadt, Germany). Images were acquired using the inverted laser scanning confocal microscope LSM 710 (Zeiss, Oberkochen, Germany).

### Enzyme-linked immunosorbent assay

TNF (R&D Systems, Minneapolis, MN), IL-12 (eBiosciences, San Diego, CA) and IL-10 (R&D Systems, Minneapolis, MN) concentration in cell supernatants was measured by Enzyme-linked immunosorbent assay (ELISA) according to the manufacturer’s instructions.

### Animal experiments

Studies were performed with 6 to 8-week-old female CB6F1 (C57BL/6xBALB/c) mice from Envigo, Scandinavia. Animals were housed in appropriate animal facilities at Statens Serum Institute. Mice (4–8/group) were immunized three times with a 3 week interval between the immunizations. Subcutaneous (s.c.) immunizations were given at the base of the tail and comprised 200 μl of 5 μg H56 protein alone or mixed with CAF01 (250 μg DDA/50 μg TDB) incorporating 0 µg, 10 µg, 20 µg or 40 µg Pam_3_Cys. Five weeks after the final vaccination the inguinal (draining) lymph nodes, the spleen and blood were harvested individually. Single cell suspensions were generated from the lymph nodes and spleens by passing the organs through a nylon mesh cell-strainer followed by washes with phosphate-buffered saline (PBS) and RPMI 1640 (Invitrogen, Carlsbad, CA, USA). The cells were re-suspended in RPMI 1640 supplemented with 10% (v/v) heat-inactivated fetal bovine serum, 5 × 10^6^ M β-mercaptoethanol, 1% (v/v) penicillin–streptomycin, 1% (v/v) sodium pyruvate, 1 mM l-glutamine, and 10 mM HEPES. The cells were counted and the concentration was subsequently adjusted to 2 × 10^6^ cells/mL. Blood samples were left overnight refrigerated in EDTA free vials and sera were obtained after centrifugation at 10,000×*g* for 5 min. Sera were stored at −20 °C until analysis.

### Intracellular flow cytometry

Lymph node cells were stimulated with 2 µg/ml H56 together with anti-CD28 (37.51; BD Biosciences) and anti-CD49d (9C10; BD Biosciences) for 1 h. Subsequently, 10 µg/well brefeldin A (Sigma-Aldrich, St. Louis, MO) and 0.7 µl/well monensin/GolgiStop (BD Biosciences Franklin Lakes, NJ) were added and cells were incubated for 5 h at 37 °C. After overnight storage at 4 °C, cells were washed in FACS buffer (1% FCS (VWR-Bie & Berntsen, Herlev, Denmark), 0.1% sodium azide (VWR, Radnor, PA) in PBS (Life Technologies, Carlsbad, CA), and stained for surface markers with 1 µg/ml anti-CD4-APC-eFlour780 (clone GK1.5) and 1 µg/ml anti-CD44-FITC (clone IM7) (both eBiosciences, San Diego, CA) for 30 min at 4 °C. Cells were washed with FACS buffer, before fixation and permeabilization using Cytofix/Cytoperm kit (BD Biosciences Franklin Lakes, NJ). Subsequently, cells were stained for intracellular cytokines with 1 µg/ml anti-IFN-γ PE-Cy7 (XMG1.2; eBiosciences, San Diego, CA), 1 µg/ml anti-TNF-PE (MP6-XT22; eBiosciences, San Diego, CA) and 1 µg/ml IL-17A for 20 min. Finally, cells were washed, re-suspended in FACS buffer, and analyzed using a FACSCanto flow cytometer (BD Biosciences Franklin Lakes, NJ) and FlowJo software version 10.

### MINCLE expression

For determining the cell surface expression of MINCLE, cells were labeled with anti-MINCLE antibodies (13 µg/ml; clone 2D12, Abnova, Taipei City, Taiwan) or IgG2a isotype control (R&D Systems, Minneapolis, MN) for 30 min at 4 °C. Cy2-conjugated goat anti-mouse antibodies (1:250, Jackson ImmunoResearch, Cambridge, UK) were used for detection. Staining was analysed using a FACSCanto flow cytometer (BD Biosciences Franklin Lakes, NJ) and FlowJo software version 10. To determine MINCLE mRNA expression CD1^+^ macrophages were generated as described above. Monocytes were isolated by plastic-adherence of freshly isolated PBMC. RNA was isolated with the RNeasy Mini Kit (Qiagen, Venlo, NL) following the manufacturer’s protocol. RNA (5 µg) was transcribed to cDNA using Oligo(dT) Primer (New England BioLabs, Ipswich, USA) followed by incubation for 10 min at 70 °C. Afterwards 4 µl 5 × First-Strand Buffer (Fermentas, ThermoFisher Scientific) and 2 µl dNTP PCR nucleotide mix (Roche, Basel, CH) were added and incubated for 5 min at 37 °C. Addition of 1 µl H-Minus RTase (Fermentas) was followed by two incubation cycles (50 min at 42 °C and 15 min at 70 °C) in the PCR thermocycler. Primers were selected as published by Ostrop et al. [13] (biomers.net GmbH, Ulm). Expression levels of MINCLE and the housekeeping gene cyclophilin A (PPIA) were determined via FastStart Essential DNA green master (Roche) using a Light Cycler Nano. Data was analyzed with supplied Light Cycler Nano software 1.0 (Roche) and GraphPad version 6.05 for Windows. ΔCT values were calculated as ΔCT = CT(PPIA) – CT(MINCLE).

### Multiplex cytokine assay

Spleen cells were re-stimulated with 2 µg/ml H56. IFN-γ, TNF and IL-17A concentrations in cell supernatants were measured by the Th1/2/17V-plex assay (MesoScaleDiagnostic, Rockville, MD) according to the manufacturer’s instructions.

### Detection of vaccine-specific antibodies

Microtiter plates (Nunc Maxisorp^™^, Roskilde, Denmark) were coated with H56 antigen (0.5 μg/ml) in carbonate-buffer pH 9.6 (SSI diagnostica, Hillerod, Denmark) overnight at 4 °C. Free binding sites were blocked with 1% (w/v) BSA (Sigma-Aldrich, St. Louis, MO) in PBS. Individual mouse sera were analyzed in duplicate in five-fold dilutions in PBS containing bovine serum albumin starting with a 100-fold dilution. Horseradish peroxidase-conjugated secondary rabbit anti-mouse IgG1 and IgG2a (Zymed) diluted 1/2000 in PBS with 1% (w/v) BSA were added for 1 h. Subsequently, antigen-specific antibodies were detected by 3,3ʹ,5,5′-tetramethylbenzidine substrate (Kem-En-Tec, Copenhagen, Denmark). 100 μl of 0.2 M sulfuric acid (VWR, Radnor, PA) was added to stop the reaction and the optical density was measured at 450 nm. The absorbance values were plotted as a function of the reciprocal dilution of serum samples.

### Statistical analysis

Statistical comparison was performed using the non-parametric Wilcoxon signed-rank test for paired samples. *p *values < 0.05 were considered significant. Calculations were performed with GraphPad Prism for Windows (version 6.05, GraphPad Software).

## Results

### Physical characterization

**Fig. 1:**
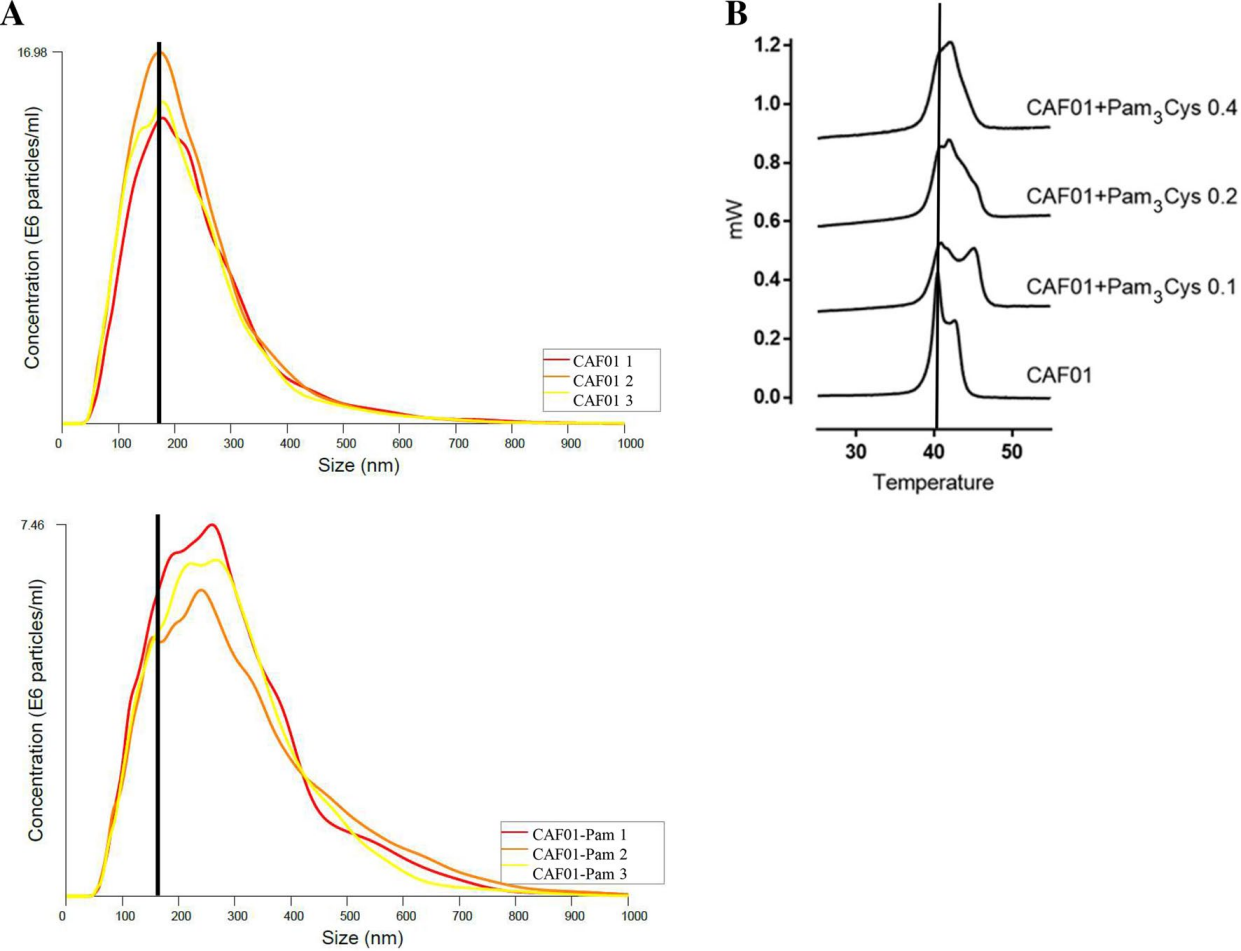
Characterization of liposomes containing Pam_3_Cys. **a** The size of liposomes was determined by Nanoparticle Tracking Analysis. Representative histograms of liposomal size distribution for CAF01 and CAF01-Pam_3_Cys are shown. Histograms were generated by the NanoSight NTA software from three independent measurements of one liposome preparation. **b** Differential scanning heat capacity curves for CAF01 liposomes incorporating increasing amounts of Pam_3_Cys. Notice that the scans have been displaced on the heat capacity axis for clarity

The size of CAF01-Pam_3_Cys liposomes was analyzed by nanoparticle tracking analysis. Empty CAF01 liposomes yielded a distinct peak at 313 ± 10.4 (Fig. 1a). CAF01-Pam_3_Cys liposomes had a similar size at all concentrations tested (0.1, 0.2 and 0.4 mg/ml). We confirmed the incorporation of Pam_3_Cys into the CAF01 liposomes by differential scanning calorimetry, showing that incorporation of Pam_3_Cys resulted in an increased phase transition temperature (*T*_start_ and *T*_max_) and a narrowing of the phase transition peak (*T*) compared to CAF01 alone (Fig. 1b). In addition, an increased enthalpy (*H*) was observed. In conclusion, the adjuvant candidate Pam_3_Cys is incorporated into H56/CAF01-liposomes yielding a homogenous liposome population of approximately 300 nm size, with only little change in the lipid bilayer characteristics.

**Fig. 2:**
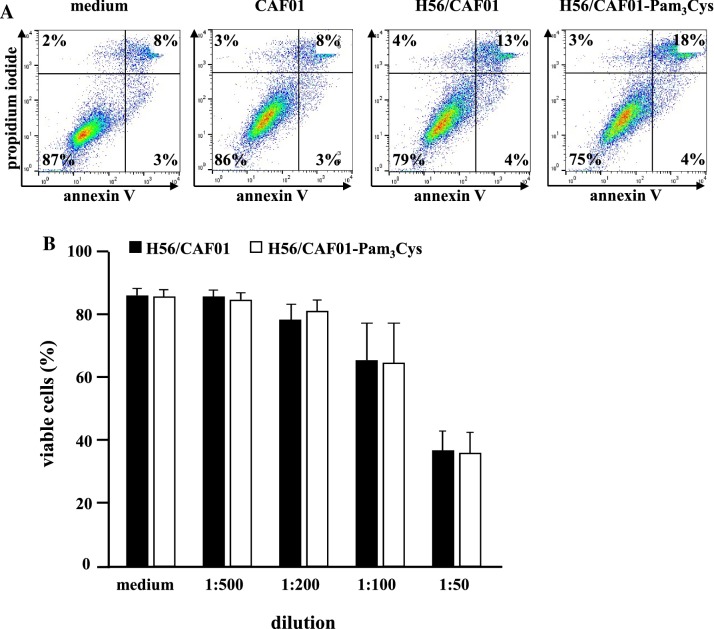
Toxicity of H56/CAF01 and H56/CAF01-Pam_3_Cys macrophages. 0.2 × 10^6^ macrophages were incubated overnight with medium alone or increasing concentrations of liposomes (DDA 1.25 mg/ml, TDB, 0.25 mg/, Pam_3_Cys 0.2 mg/ml, H56 0.1 mg/ml). The percentage of viable cells was determined by annexin V-FITC and propidium iodide staining. **a** Flow cytometry data of one representative donor for the medium control and liposomes diluted 1:200 are shown. **b** The graph gives the average percentage ± SD of viable cells (*n* = 5)

In vitro studies: Before performing functional experiments, we determined whether the liposome preparations exert toxicity against primary human cells. We selected CD1^+^ macrophages as target cells, since the long-term objective of this project is the delivery of mycobacterial lipid antigens, which require group1 CD1 molecules for antigen presentation. Macrophages were incubated with liposomes at increasing concentrations and the percentage of necrotic and apoptotic cells was determined by annexin-V-FITC/ propidium iodine staining after 18 h of incubation (Fig. 2). All liposomal preparations tested moderately reduced the viability of macrophages from 10% (medium control) to 17% (H56:CAF01). Importantly, the inclusion of Pam_3_Cys into H56:CAF01 liposomes had no significant effect on the viability of macrophages at 1:500-, 1:200-, 100- or 1:50 dilution (Fig. 2b). However, the highest liposome concentration (1:50 dilution) markedly reduced the viability of macrophages to below 40% and was omitted from the following experiments.

**Fig. 3:**
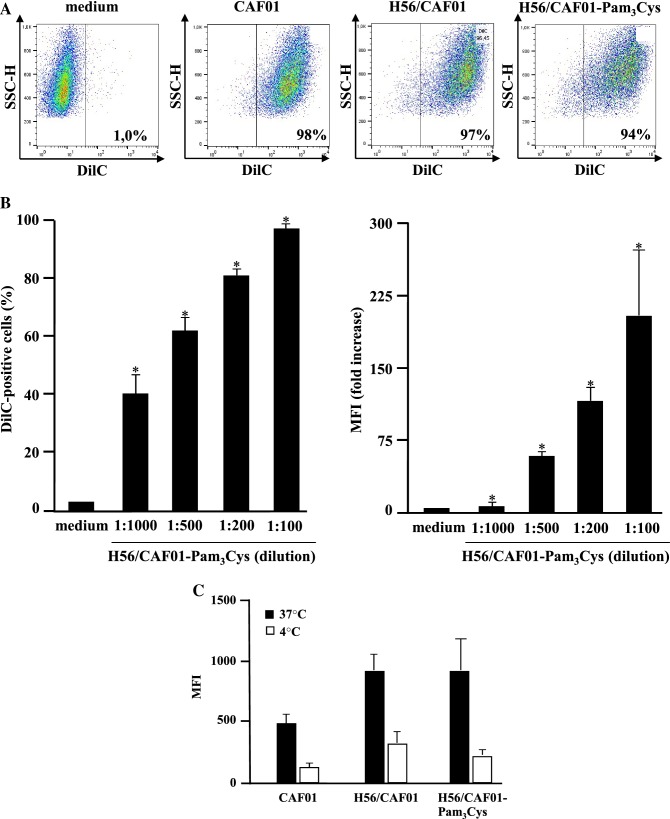

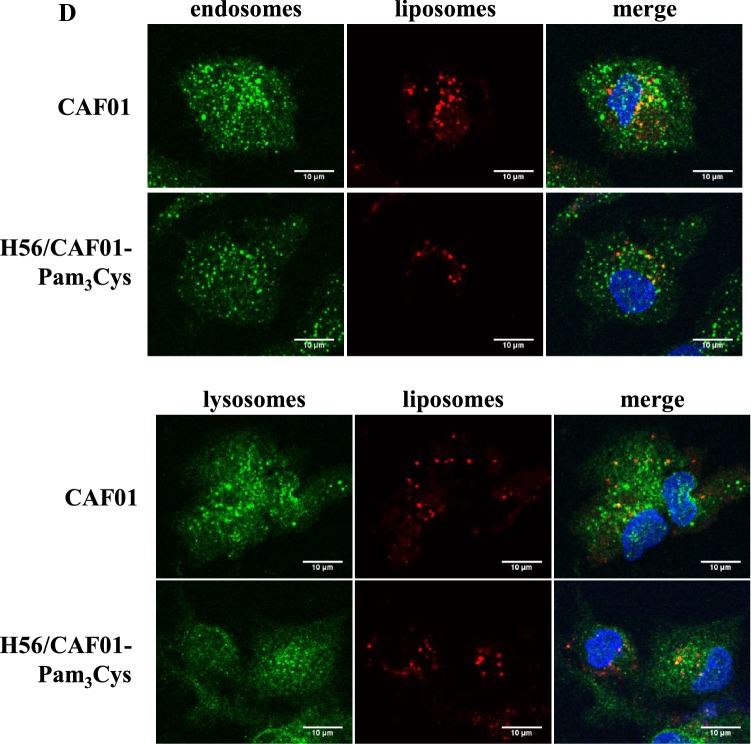
Uptake of DilC-tagged H56/CAF01-Pam_3_Cys by human macrophages. **a** and **b** 0.2 × 10^6^ macrophages were incubated with medium alone or increasing concentrations of DilC-labeled liposomes (DDA 1.25 mg/ml, TDB, 0.25 mg/, Pam_3_Cys 0.2 mg/ml, H56 0.1 mg/ml). After 16 h, cells were stained for MHC class II expression. The number of cells staining positively for fluorescent liposomes and the MFI were detected by flow cytometry. **a** Figure shows flow cytometry data of one representative donor for the medium control and liposomes diluted 1:200. **b** Graphs give the average percentage ± SD and the mean fluorescence intensity (MFI) ± SD of MHCII^+^/DilC^+^ cells of five independent donors. Fold change was calculated as ratio to media control cells. *p < 0.05 as compared to medium control (non-parametric Wilcoxon–Mann–Whitney Test for paired samples). **c** Effect of inhibiting endocytic *p*athways on the MFI. 0.2 × 10^6^ CD1^+^ macrophages were incubated overnight with DilC-labeled liposomes (1:200) at 37 °C or 4  °C. The graph compares the average MFI ± SD of five independent donors. **d** Intracellular localization of fluorescently-labeled H56/CAF01-Pam_3_Cys in macrophages. 0.1 × 10^6^ macrophages were incubated overnight with DilC-labeled liposomes (1:100). Endosomes or lysosomes were labeled using an anti-EEA1 or anti-LAMP1 antibody, respectively. Cell nuclei were stained with DAPI. Images were acquired using an inverted laser scanning confocal microscope. Representative areas of three different donors are shown. Endosomes or lysosomes are depicted in green, DilC-labeled liposomes in red, cell nuclei in blue. Yellow color indicates co-localization of liposomes with endosomes/lysosomes. Scale bar is 10 µm

Antigen presentation and T-cell activation requires efficient uptake of the antigen and delivery into endosomal/lysosomal compartments. To quantitate the uptake, liposomes were labeled with the red lipophilic tracer DilC and phagocytosis was determined by flow cytometry after overnight incubation (Fig. 3). At a dilution of 1:100, almost all macrophages (94–98%) stained positively for the DilC, independently of the composition (CAF01, H56/CAF01, H56/CAF01-Pam_3_Cys). Since this concentration moderately increases the toxicity (Fig. 2) we investigated whether an equally efficient uptake could be achieved with lower liposome concentrations. The number of cells as well as the mean fluorescence intensity (MFI) increased in a dose dependent manner and reached > 80% at the non-toxic dilution of 1:200 (Fig. 3b). The staining was significantly decreased when cells were incubated 4 °C (Fig. 3c), suggesting that the liposomes are actively internalized rather than only associated with the outer cell membrane. To further support and extend this finding we incubated macrophages with DilC-tagged CAF01 or H56:CAF01-Pam_3_Cys liposomes and co-labeled the endosomal (EEA-1) and lysosomal (LAMP-1) compartments. Analyses by confocal laser microscopy demonstrated that (1) liposomes were localized intracellularly, (2) liposomes co-localized with endosomes and lysosomes to a similar extent and (3) inclusion of the vaccine H56 antigen and the adjuvant Pam_3_Cys did not significantly modulate the uptake or intracellular localization of the liposomes into the macrophages. In conclusion, CAF01 ± Pam_3_Cys liposomes are efficiently phagocytosed by primary human macrophages and shuttled into the endosomal/lysosomal compartment.

**Fig. 4:**
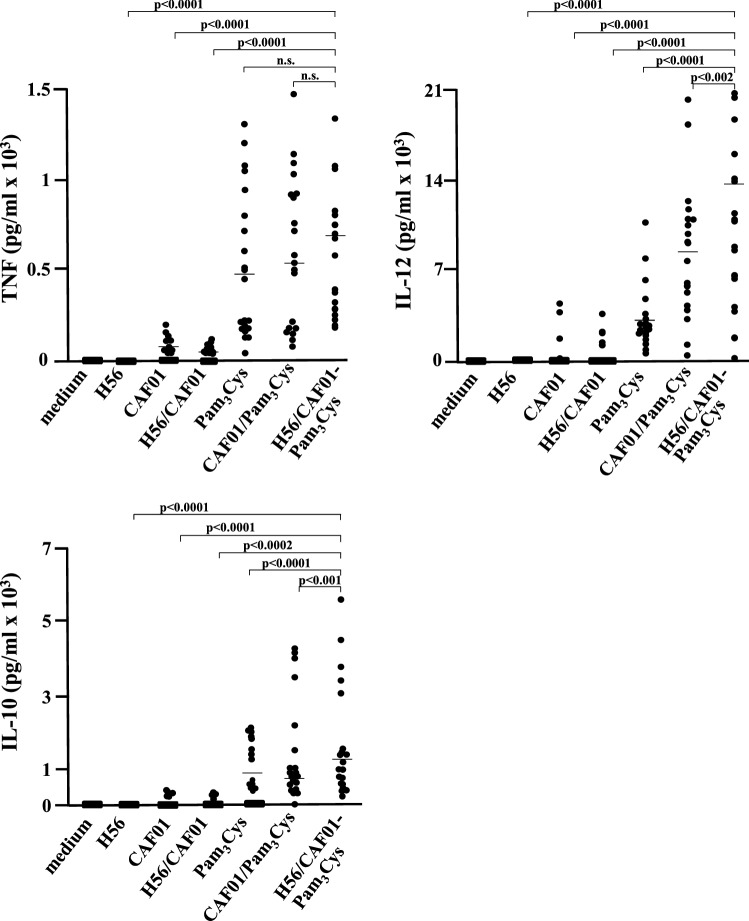
Cytokine release by H56/CAF01-Pam_3_Cys stimulated macrophages. 0.2 × 10^6^ macrophages were incubated overnight with H56 (0.5 µg/ml), CAF01 (1:200), Pam_3_Cys (1 µg/ml), H56/CAF01 liposomes (DDA 1.25 mg/ml, TDB, 0.25 mg/, H56 0.1 mg/ml) or H56/CAF01-Pam_3_Cys liposomes (DDA 1.25 mg/ml, TDB, 0.25 mg/, Pam_3_Cys 0.2 mg/ml, H56 0.1 mg/ml) (1:200) as indicated. TNF, IL-12 and IL-10 release were measured by sandwich ELISA. Dots represent individual values and lines indicate median values

**Fig. 5:**
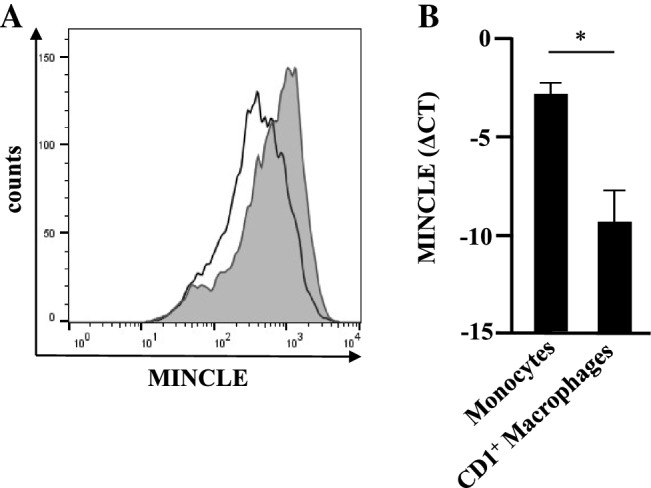
MINCLE expression by monocytes and macrophages. **a** 0.5 × 10^6^ macrophages were stained with anti-MINCLE antibodies (5 µl). The histogram depicts the isotype control (IgG2a) and MINCLE-staining (gray). The graph presents one representative result of five donors with similar result. **b** mRNA of 3 × 10^6^ monocytes or macrophages was isolated and MINCLE expression quantified using RT-PCR. Higher values indicate higher relative expression levels. The figure shows mean mRNA expression levels ± SD from all three donors tested

To investigate whether the interaction of macrophages and liposomes triggers the release of immune-modulatory cytokines we measured the release of TNF, IL-12 and IL-10 after overnight incubation with liposomes (1:200). Un-treated macrophages, H56, empty CAF01-liposomes or liposomes containing H56 induced no or minute amounts of the three cytokines measured (Fig. 4). Pam_3_Cys increased the antigen-specific release of TNF, IL-12 and IL-10 by as compared to H56/CAF01 in all donors investigated (*n* = 20; *p* < 0.0001) (Fig. 4). IL-12 and IL-10 release were also significantly enhanced by H56/CAF01/Pam_3_Cys as compared to Pam_3_Cys alone or CAF01/Pam_3_Cys. These results demonstrate that inclusion of the Toll-like receptor agonist Pam_3_Cys into liposomes induces the release of immune-modulatory cytokines, which could promote macrophage activation, chemotaxis and ultimately vaccine-antigen specific T-cell activation in vivo. Since the level of cytokine release triggered by CAF01 alone was moderate, we speculated that the major receptor for CAF01, MINCLE [13] is absent in CD1^ + ^macrophages used in our experiments. To address this question, we compared the mRNA expression for MINCLE in freshly purified monocytes and CD1^+^ macrophages by qPCR. In all three donors MINCLE mRNA-levels were significantly lower as compared to monocytes derived from the same donors (Fig. 5a). Nevertheless, MINCLE was reproducibly (*n* = 5) detectable at low levels on the cell surface of CD1^+^ macrophages (Fig. 5b). Therefore, the moderate expression of the trehalose-6-6-dimycolate receptor MINCLE provides a possible explanation for the limited release of TNF, IL-12 and IL-10 in our experiments.

**Fig. 6:**
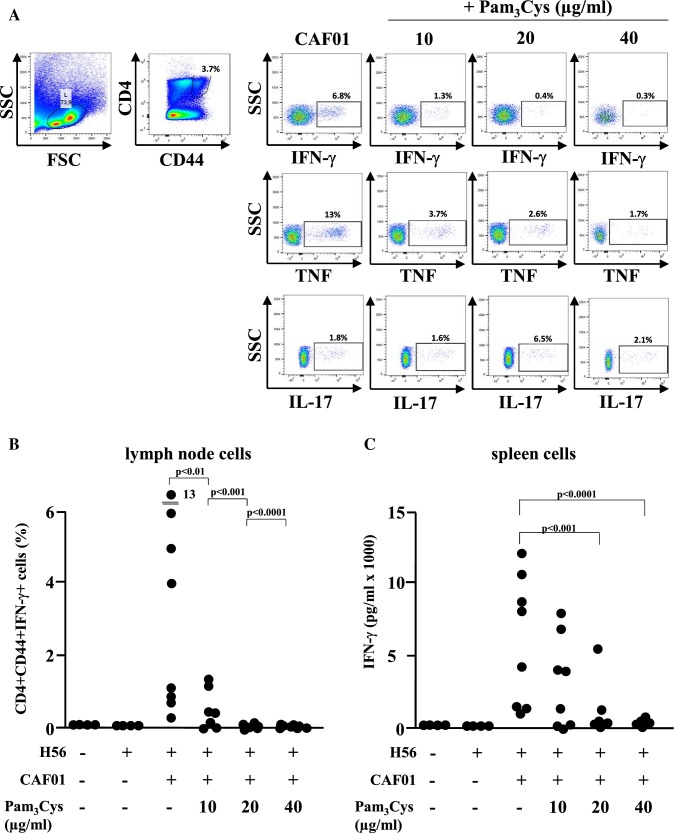

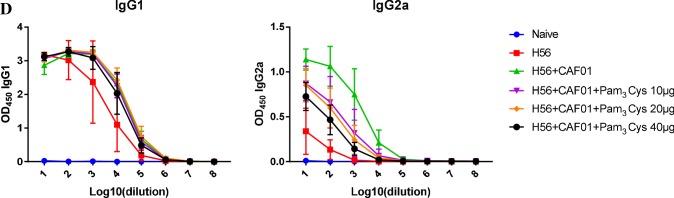
IFN-γ production upon immunization of mice with H56 antigen adjuvanted with CAF01-Pam_3_Cys. CB6F1 mice (*n* = 4–8) were immunized s.c. three times at 3-week intervals with H56 alone or adjuvanted with CAF01 or CAF01-Pam_3_Cys. Five weeks after the last immunization lymph node cells, spleen cells and blood were harvested. Lymph node cells were subjected to intracellular flow cytometry after re-stimulation with H56 antigen (5 µg/ml). **a**. CD4^+^, CD44^+^ lymphocytes were gated and further analysed for the expression of IFN-γ, TNF or IL-17A. The graph shows the gating strategy for one representative animal **b**. The graph shows the percentage of activated T cells (CD4^+^ CD44^+^) producing IFN-γ. Data points represent individual mice. **c** IFN-γ release from spleen cells re-stimulated with H56 (5 µg/ml) was measured using MSD cytokine assay. The individual results of all mice are shown. **d** Presence of antigen-specific IgG1 and IgG2a in serum was analyzed by indirect ELISA

**Fig. 7:**
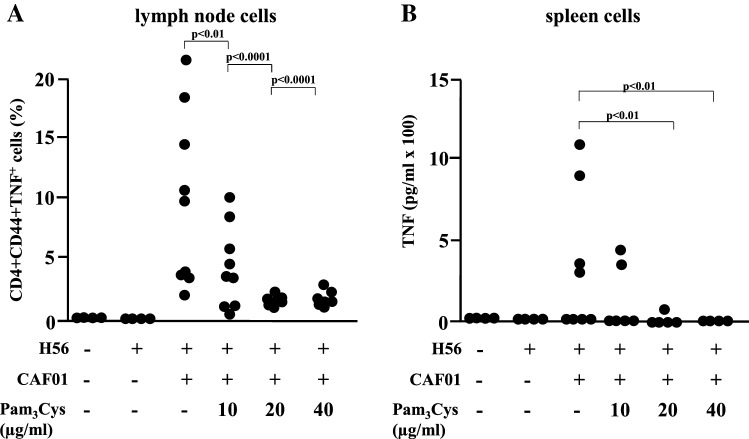
Influence of the immunization of mice with H56 antigen adjuvanted with CAF01-Pam_3_Cys on the expression of TNF. CB6F1 mice were immunized s.c. three times at 3 week intervals. Lymph node cells, spleen cells and blood were harvested five weeks after the final immunization. **a** Lymph node cells were in vitro re-stimulated with H56 antigen (5 µg/ml) and the percentage of antigen-specific CD4^+^ CD44^+^ cells expressing TNF was determined by intracellular flow cytometry. Dots depict individual mice (*n* = 4–8). **b** TNF release by spleen cells upon re-stimulation with H56 antigen (5 µg/ml) was measured by MSD cytokine assay. The graph shows the individual results of all mice tested (*n* = 4–8)

**Fig. 8:**
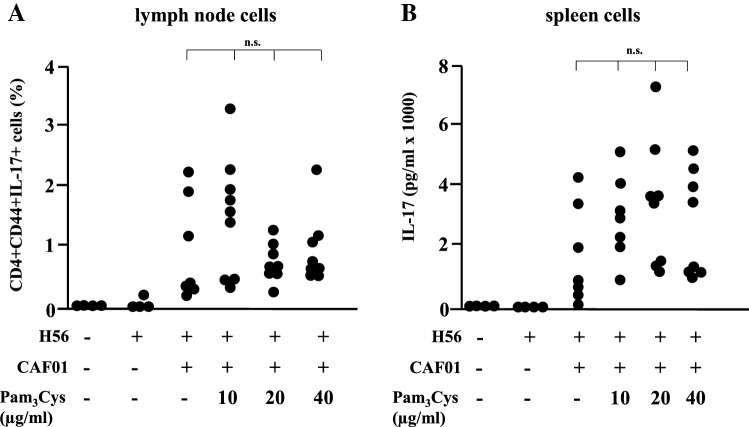
Effect of vaccinating mice with the antigen H56 adjuvanted with CAF01-Pam_3_Cys on the expression of IL-17A. CB6F1 mice were immunized s.c. three times at 3 week intervals with H56 alone or adjuvanted with CAF01 or CAF01-Pam_3_Cys. Lymph node cells, spleen cells and blood was isolated five weeks post-third immunization. **a** Percentage of CD4^+^ CD44^+^ lymph node cells expressing IL-17A after ex vivo H56 re-stimulation (5 µg/ml) were determined by intracellular flow cytometry. Data points represent individual mice (*n* = 4–8). **b** Concentration of IL-17A in supernatants of spleen cells re-stimulated with H56 antigen (5 µg/ml) was determined by MSD cytokine assay. The graph gives the individual results of all mice

In vivo studies: To test this hypothesis, six groups of mice (7–8 mice per group) were immunized three times on day 0, 21 and 42, with H56/CAF01 containing three different concentrations of Pam_3_Cys (0.1, 0.2 and 0.4 mg/ml). Control groups were immunized with H56 alone or left untreated. Two weeks after the last immunization, cell lysates were prepared from lymph nodes and spleens, re-stimulated in vitro with the vaccine antigen H56 and analyzed for cytokine production by ELISA and intracellular flow cytometry. Immunization with H56 alone failed to induce IFN-γ production by activated (CD44-positive), CD4-positiv T-lymphocytes (Fig. 6a, b) in re-stimulated immune cells as determined by intracellular cytokine staining (lymph node cells; Fig. 6b) or ELISA (spleen cells: Fig. 6c). Delivery of the vaccine antigen with CAF01 triggered profound IFN-γ production, whereas inclusion of the adjuvant Pam_3_Cys into the CAF01 liposomes reduced (10/20 µg/dose) and even diminished (40 µg/dose) the induction of the IFN-γ producing cells (Fig. 6c). Even though there was a considerable variability between individuals animals the findings were statistically significant. In agreement with this pattern, the concentration of the IFN-γ dependent IgG2a-specific anti H56 antibodies in the serum was reduced in a dose-dependent manner by addition Pam_3_Cys into the liposomes (Fig. 6d), while the concentration of IFN-γ-independent IgG1 remained unaffected. Similarly, the frequency of TNF-producing cells in the lymph node and the TNF-release by H56-stimulated spleen cells was almost completely diminished in mice immunized with Pam_3_Cys containing liposomes (Fig. 7). This was not due to complete deactivation of the cytokine synthesis machinery, because the expression and release of IL-17A remained unaffected by addition of Pam_3_Cys into the CAF01 liposomes. Taken together these results demonstrate that the inclusion of the adjuvant Pam_3_Cys into CAF01 has profound effects on the immunogenicity in vivo. Specifically, the antigen-specific production of IFN-γ and TNF are reduced, while the production of IL-17A is maintained. Therefore, the adjuvant Pam_3_Cys has differential effects on the vaccine-induced cytokine release in vivo and biases the immune response towards a Th17 specific immune response (Fig. 8).

## Discussion

Pam_3_Cys is the biologically active component of the mycobacterial 19 kDa lipoprotein of mycobacteria. By interaction with Toll like receptor 2/1 it induces an intracellular signaling pathway resulting in increased synthesis of the antimicrobial peptide cathelicidin and killing of intracellular *Mtb* [10]. The present study exploited the use of the liposome based adjuvant system CAF01 to deliver Pam_3_Cys and showed that Pam_3_Cys has profound effects on the elicited immune responses both in vitro and in vivo. Using the *Mtb*-derived multi-subunit vaccine H56, which is currently evaluated in clinical trials we demonstrate that the inclusion of Pam_3_Cys into CAF01 promotes the release of cytokines, which are associated with protection against tuberculosis in vitro (TNF, IL-12). In vivo the injection of H56/CAF01-Pam_3_Cys in mice suppresses the antigen specific Th1 response but maintains the Th17 response induced by CAF01. These findings demonstrate that Toll like receptor 2/1 agonist modulate vaccine-mediated immune responses. The differential effects in human macrophages and in mice give a note of caution for interpreting results based on one species only.

To investigate whether inclusion of Pam_3_Cys into liposomes modulates the cytokine release by macrophages, we measured the secretion of three cytokines that are essential for shaping the immune response in tuberculosis, TNF, IL-12 and IL-10. In humans, the major function of TNF in tuberculosis is the orchestration of cellular movement required for the formation of tuberculous granulomas [14]. Neutralization of TNF during the course treatment of autoimmune disorders leads to disruption of the granulomas and reactivation of latent infection [15]. In addition anti TNF treatment reduces the frequency of CD8-positive T cells in the peripheral blood which express the antimicrobial peptide granulysin, which by inference correlates with an increased risk to develop active tuberculosis [14]. The key role of IL-12 in protection against tuberculosis in humans has become evident by experiments of nature in which patients with autosomal recessive, complete IL-12p40 or IL-12Rß1 deficiency typically suffer from invasive mycobacterial infections [16]. Functionally, IL-12 is required for supporting IFN-γ-mediated activation of macrophages [17]. The role of IL-10 in human tuberculosis ranges broadly from anti to pro-inflammatory functions. IL-10 hampers antigen-specific T-cell responses, affects apoptosis of infected macrophages, interferes with antigen presentation, enhances mycobacterial survival in macrophages [18] and promotes the differentiation of macrophages to dendritic cells [19, 20]. At first glance, this suggests that IL-10 favors the pathogen more than the host does. However, a hallmark of active tuberculosis infection is tissue destruction by immunopathology mediated by proliferating, cytokine-releasing and cytotoxic T-lymphocytes. In this respect, IL-10 has an important function in balancing T-cell activation and limiting tissue destruction after the initial immunological burst. Taken together the inclusion of Pam_3_Cys into H56/CAF01 skews the cytokine profile of macrophages towards a protective (TNF, IL-12) and anti-inflammatory pattern (IL-10). This suggests that TLR 2/1 ligands have the potential to support vaccine efficacy without triggering immunopathology by inflammation. In contrast, immunization of mice with H56/CAF01-Pam_3_Cys suppressed *Mtb*-specific Th1 polarization illustrating that human macrophages are not an appropriate experimental system to predict immune responses in an in vivo murine model. In addition, the route of vaccination may have favored the suppression of Th1 responses. Cutaneous immunization as employed in our experiments, induces the infiltration of CCR2^+^ monocytes to the skin draining lymph nodes and inhibits priming of Th1 responses [21]. Similarly, BCG, which expresses abundant TLR2/1 agonists, induces Th2 skewed T-cell responses when administered intradermally as opposed to intravenous application, where Th1-responses predominate [21]. Furthermore, intravenous BCG vaccination provides superior protection against *Mtb*-challenge than intradermal or aerosol application in non-human primates [22]. From a practical point of view aerosol-delivery of vaccines avoids needles and associated side effects such as infections especially in resource-limited environments. The aerosol route of mycobacterial vaccine antigens has been shown to be safe and promote local immunity in the lung in a phase I randomized clinical trial [23]. Species-dependent differences and the effect of the route of antigen delivery are just two obstacles exemplifying the difficulty of developing in vitro models for predicting in vivo immune responses. Our conflicting results on the immunogenicity of a TLR-agonist adjuvanted mycobacterial antigen in human macrophages and in mice illustrates these challenges. Nevertheless, we believe it is relevant to perform side-by-side comparisons of human and murine experimental models to enhance our understanding on species-specific immunity and instruct translational research. The mechanism for the reduced Th1 response could be related to the route of vaccine administration. The cutaneous delivery of TLR2-agonist induces the infiltration of CCR2^+^ monocytes to the skin draining lymph nodes and inhibits priming of Th1 responses in mice [21]. In contrast to Th1-polarization, the *Mtb*-specific IL-17A-release was not affect by Pam_3_Cys. IL-17A is essential for an efficient vaccination against tuberculosis [24, 25], because it is part of the vaccine-induced polyfunctional T-cell response [26, 27] and contributes to the formation of lung follicle formation by inducing CXCL-13 production [28]. While our in vivo-findings offer relevant insights into the contribution of Pam_3_Cys to the immunogenicity of the vaccine candidate H56, the translation of immunological findings from mice to humans needs to be interpreted with caution. In the context of the experiments presented here it is noteworthy, that mice do not have a vitamin D response element and hence are unable to initiate the Toll-like receptor-mediated, cathelicidin dependent antimicrobial pathway [29].

Three critical questions remain unresolved due to technical limitations, but will be addressed in future experiments. First, the evaluation of human T-cell responses to H56/CAF01-Pam_3_Cys is dependent on the identification and recruitment of MHC-matched donors and more importantly by the in vitro toxicity of DDA-containing liposomes at concentrations required for T-cell activation. Secondly, it remains speculative whether Pam_3_Cys increases the protective efficacy of the H56:CAF01 vaccine in a challenge model of murine tuberculosis and how the route of immunization influences immunogenicity. Finally, in vitro studies using murine macrophages will be important to understand the cellular mechanisms for the suppression of Th1 response in vivo.

Taken together our study demonstrates that the Toll like receptor 2/1 agonist Pam_3_Cys has a significant impact on the immunogenicity of the tuberculosis vaccine candidate H56:CAF01 in vitro (primary human macrophages) and in vivo (mice). While Pam_3_Cys promotes activation of human macrophages, Th1-polarization in immunized mice is reduced, whereas the Th17 response, which is critical for efficient initiation of a vaccine response, is maintained. These findings provide technical and experimental proof of principle that the immunogenicity of vaccines can be manipulated by the inclusion of adjuvants, which address the innate immune response.
